# Supplementary figures and images for: Variables appended to ABS frames: Has their data quality improved?
Source: PLoS One. 2022 Nov 2;17(11):e0269110. doi: 10.1371/journal.pone.0269110 (PMC9629543; doi:10.1371/journal.pone.0269110)

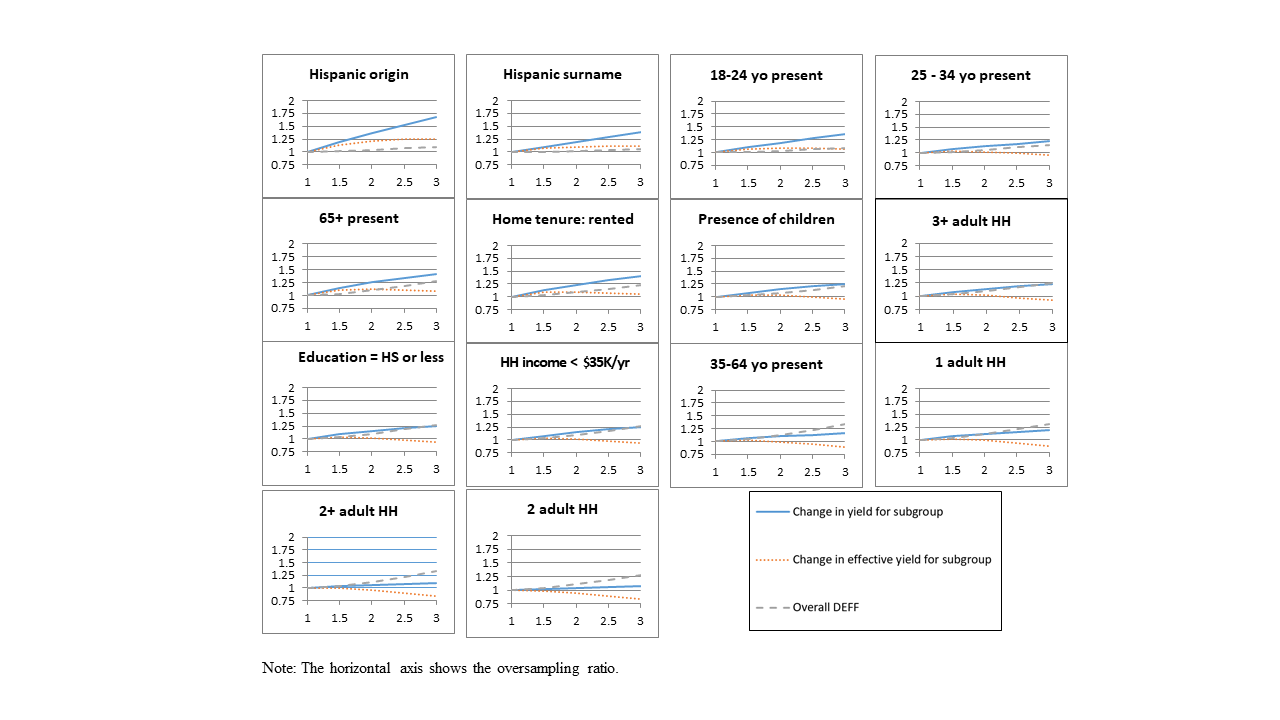

Supplement: S1 Fig — Note: The horizontal axis shows the oversampling ratio. (TIF) [file pone.0269110.s001.tif]
