# Supplementary material for: Variables appended to ABS frames: Has their data quality improved?
Source: PLoS One. 2022 Nov 2;17(11):e0269110. doi: 10.1371/journal.pone.0269110 (PMC9629543; doi:10.1371/journal.pone.0269110)

**Online Appendix for: “Variables Appended to ABS Frames: Has Data Quality Improved?”**

**Questionnaire Items from 2017 Health Information National Trends Survey Cycle 1 (HINTS5)**

**Number and age of household adults:**

The number of household adults is calculated in three ways on the HINTS survey and a reconciliation process is implemented to come up with the final response in situations where the measures do not align. The presence of household members in certain age groups was derived from the 3rd measure (the household roster):

*Method 1 – Questions from the respondent selection page:*

1. Is there more than one person age 18 or older living in this household?

Yes

No **GO TO A1 on the next page**

2. Including yourself, how many people age 18 or older live in this household?

|  |
| --- |

M*ethod 2 – Subtracting household members to household children*

O13. Including yourself, how many people live in your household?

|  |  | Number of people |
| --- | --- | --- |

O15. How many children under the age of 18 live in your household?

|  |  | Number of children under 18 |
| --- | --- | --- |

*Method 3 – household roster (used for number of adults & presence of adults in age groups):*

O14. Starting with yourself, please mark the sex, and write in the age and month of birth for each adult 18 years of age or older living at this address.

|  | Sex | Age | Month Born (01-12) | |
| --- | --- | --- | --- | --- |
| **SELF** | Male  Female | \|  \|  \|  \| \| --- \| --- \| --- \| | \|  \|  \| \| --- \| --- \| |  |
| Adult 2 | Male  Female | \|  \|  \|  \| \| --- \| --- \| --- \| | \|  \|  \| \| --- \| --- \| |  |
| Adult 3 | Male  Female | \|  \|  \|  \| \| --- \| --- \| --- \| | \|  \|  \| \| --- \| --- \| |  |
| Adult 4 | Male  Female | \|  \|  \|  \| \| --- \| --- \| --- \| | \|  \|  \| \| --- \| --- \| |  |
| Adult 5 | Male  Female | \|  \|  \|  \| \| --- \| --- \| --- \| | \|  \|  \| \| --- \| --- \| |  |

**Questionnaire Items from 2011 National Household Education Survey (NHES) Field Test**

**Educational attainment:**

What is the highest grade or level of school that <respondent> completed?

□ 8^th^ grade or less

□ High school, but no diploma

□ High school diploma or equivalent (GED)

□ Vocational diploma after high school

□ Some college, but no degree

□ Associate’s degree (AA, AS)

□ Bachelor’s degree (BA, BS)

□ Some graduate or professional education, but no degree

□ Master’s degree (MA, MS)

□ Doctorate degree (PhD, EdD)

□ Professional degree beyond bachelor’s degree (MD, DDS, JD, LLB)

**Home tenure:**

Is this house…

*Mark* **☒** *ONE only.*

□ Owned or being bought by someone in this household,

□ Rented by someone in this household, or

□ Occupied by some other arrangement?

**Household income:**

Which category best fits the total income of all persons in your household over the past

12 months?

*Include your own income.*

*Include money from jobs or other earnings, pensions, interest, rent, Social Security*

*payments, and so on.*

□ $0 to $10,000

□ $10,001 to $20,000

□ $20,001 to $30,000

□ $30,001 to $40,000

□ $40,001 to $50,000

□ $50,001 to $60,000

□ $60,001 to $75,000

□ $75,001 to $100,000

□ $100,001 to $150,000

□ $150,001 or more

**Presence of children:**

A series of questions were used to determine whether children resided in each household. The derivation included the following questions:

1. Are there any youth or children age 20 or younger living in this household?

*Do not include those living in college housing.*

□ Yes

□ No

2. How many youth or children age 20 or younger live in this household?

|  |  | number age 20 or younger |
| --- | --- | --- |

3. How old is this child in years?

□ Mark for babies less than 1 year old

|  |  | age in years |
| --- | --- | --- |

**Questionnaire Items from 2017 National Household Travel Survey (NHTS)**

**Ethnicity:**

Are you of Hispanic or Latino origin?

□ Yes, Hispanic or Latino

□ No, not Hispanic or Latino

**Educational attainment:**

What is the highest grade or year of school you completed?

□ Less than a high school graduate

□ High school graduate or GED

□ Some college or Associates degree

□ Bachelor’s degree

□ Graduate degree or professional degree

**Home tenure:**

Do you own or rent your home?

□ Own

□ Rent

□ Other, please specify

**Household income:**

Please identify which category represents your total household income, before taxes, for last year. Include income from sources such as wages and salaries, income from a business or a farm, Social Security, pensions, dividends, interest, rent and any other income received for all household members.

□ Less than $10,000

□ $10,001 to $14,999

□ $15,000 to $24,999

□ $25,000 to $34,999

□ $35,000 to $49,999

□ $50,000 to $74,999

□ $75,000 to $99,999

□ $100,000 to $124,999

□ $125,000 to $149,999

□ $150,000 to $199,999

□ $200,000 or more

**Presence of children:**

Age of each person in the household is collected and whether or not children are in the household is derived by flagging the household if there is any household member under 18. The question text and an example of the matrix for Person 1 is given below.

In order to understand how people get from place to place, it’s important for us to know who currently lives in your household. Including yourself, please write in the first name/nickname, age, and gender of each person currently living in your household, including children and newborn babies.


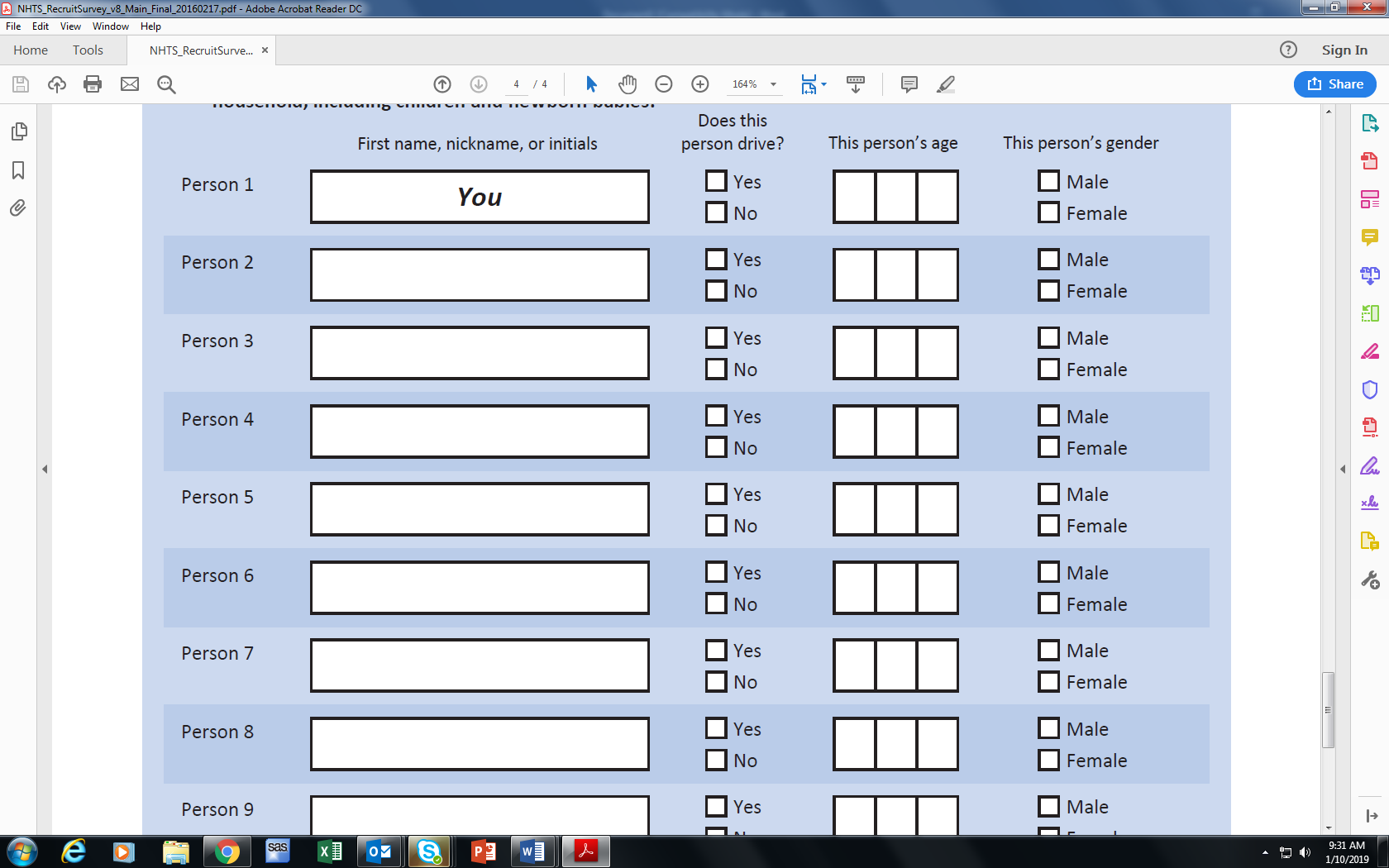

Supplement: S1 Appendix — (DOCX) [file pone.0269110.s002.docx]
