# Supplementary material for: Variables appended to ABS frames: Has their data quality improved?
Source: PLoS One. 2022 Nov 2;17(11):e0269110. doi: 10.1371/journal.pone.0269110 (PMC9629543; doi:10.1371/journal.pone.0269110)
Supplement: Attachment — Submitted filename: Comment PLOS ONE_ PONE-D-21-31646 - Sureeporn.pdf [file pone.0269110.s004.pdf]

Title: **Variables Appended to ABS Frames: Has Data Quality Improved?**

Manuscript no. PONE-D-21-31646

Reviewer: Sureeporn Punpuing, Ph.D

General:

- This study is useful for researchers who deal with quantitative survey data – particularly on the survey sampling design, data analysis and the interpretation of the findings.
- It presents the original research. However, there is a level of plagiarism (based on the 'Turn It In', please see results in an attached file).
- Results reported have not been published elsewhere. Although, there are number of similar published publications, especially the reference # 4 that the authors would like to follow up with an improvement of the appended data over time (line 96).
- Mathematics formula and data analysis performed well in an appropriate manner, but it need references for methods and formulas.
- Conclusions are clearly drawn from the results.
- The data analysis of this manuscript was based on the secondary survey data sets, and appended data from a vendor. The research meets all applicable standards for the ethnics of research integrity.

Specific:

- **Introduction:**
  The rationale of this study should be strengthened, and research gaps should be clearly identified. These would be aligned with objectives and methodology.
- **Data and methods:**
  The authors end up with using three data sets (NHTS, HINTS, ACS), but under in Table 1, the authors provide 2 detail data sets, while the third dataset was not directly use for data analysis was presented. Why did the authors give the NHES detail? Please clarify.
- **Availability and quality of appended demographic information:**
  - Please define 'demographic information' used in this study. Normally, demographers define demographic variables as age, sex, ethnicity/race of the population.
  - Fig.1 and Fig.2, please label x-axis and y-axis.
  - Lines 144-145: descriptive, please check its consistency with the figures in Table 2.
- **Efficiency of using appended demographic variables for oversampling**
  - "Oversampling" was not mentioned in the rationale- please add argument why researcher should examine efficiency of using appended data for oversampling.
  - Table 3: please clarify why '18-24 present' was not highlighted in the table, while its optimum oversampling rate =2.3 (above 2)

- Line 214; according to the results, please clarify why the household income is the less promising variable.
- The American Community Survey (ACS), please clarify why didn't the authors mentioned it at the beginning (in the data and methods session).
- Suggest: that the authors add references for each formula, and show a sample calculation e.g for the first figures in Tables 2 & 3.

- **Discussion:**
  The authors should add work more on the strengthen and limitation of this study, and how it support or contrast with the existing knowledge that was reviewed in the introduction. At this stage, there is almost no discussion in this session.
- **References:**
  Only 15 references were mentioned, while references related to methods and calculation should be added.

****
