# Supplementary material for: Variables appended to ABS frames: Has their data quality improved?
Source: PLoS One. 2022 Nov 2;17(11):e0269110. doi: 10.1371/journal.pone.0269110 (PMC9629543; doi:10.1371/journal.pone.0269110)
Supplement: Attachment — Submitted filename: Response to Reviewers.docx [file pone.0269110.s005.docx]

# Response to Reviewers

**Comments to the Author**

1. Is the manuscript technically sound, and do the data support the conclusions?

The manuscript must describe a technically sound piece of scientific research with data that supports the conclusions. Experiments must have been conducted rigorously, with appropriate controls, replication, and sample sizes. The conclusions must be drawn appropriately based on the data presented.

Reviewer #1: Yes

Reviewer #2: Partly

2. Has the statistical analysis been performed appropriately and rigorously?

Reviewer #1: Yes

Reviewer #2: Yes

3. Have the authors made all data underlying the findings in their manuscript fully available?

The [PLOS Data policy](http://secure-web.cisco.com/1yRMjc4EdK72_LMPovC7rknlDwh772sDqZImzdLnP0tVPNnEyi2XtiBpWtzJ6o_IT9N7eyrs-RrLmE-30CmZucZpG-B72lnXEKd5etallsTu08g036WF-OAgoHx8zgFAhHp9WOwvuXlw1UrxIML9nCKxqbdbn2XLoUnP8qNc1g3qcObfnB_RNu2Xno-oMpAAp7HtPqXlBLfI8mx8AmM2LOPCD9l-M48wZROfqbgYwjXdUBbxeLTuGsOyhHTVQ_knD/http%3A%2F%2Fwww.plosone.org%2Fstatic%2Fpolicies.action%23sharing) requires authors to make all data underlying the findings described in their manuscript fully available without restriction, with rare exception (please refer to the Data Availability Statement in the manuscript PDF file). The data should be provided as part of the manuscript or its supporting information, or deposited to a public repository. For example, in addition to summary statistics, the data points behind means, medians and variance measures should be available. If there are restrictions on publicly sharing data—e.g. participant privacy or use of data from a third party—those must be specified.

Reviewer #1: No

Reviewer #2: No

4. Is the manuscript presented in an intelligible fashion and written in standard English?

PLOS ONE does not copyedit accepted manuscripts, so the language in submitted articles must be clear, correct, and unambiguous. Any typographical or grammatical errors should be corrected at revision, so please note any specific errors here.

Reviewer #1: Yes

Reviewer #2: Yes

5. Review Comments to the Author

Please use the space provided to explain your answers to the questions above. You may also include additional comments for the author, including concerns about dual publication, research ethics, or publication ethics. (Please upload your review as an attachment if it exceeds 20,000 characters)

Reviewer #1: Question 3 above befuddled me. The paper goes to great length to indicate that the proprietorial vendors of ABS frames do not always fully explain the nature and methods of their data, so it is not likely that this paper could make all underlying data fully available. But it seems that they have made everything available that was within their control.

This is a very useful study as far as it goes. My comments are in the nature of pushing the boundaries further. Being from overseas, I thought the authors might have helped the foreign readers to understand how the ABS might differ between US, UK, Canada, Australia, and European countries. I know that many Asian countries would struggle to attain the levels of coverage of the USPS.

***Author response:***

***Given that all of the underlying data were collected in the US using the ABS frame based on USPS lists, our research is currently limited to US only. We have clarified this in the manuscript abstract (it is already detailed in the Introduction).***

Hispanic and non-Hispanic is a category I know from the US Census, but it wasn't clear why the vendors did not append questions on race.

***Author response:***

***We used ABS appended data that we were able to match to variables that were available in the studies we evaluated. Hispanic/Non-Hispanic were available from both the vendor and the surveys, but, as the reviewer noted, race variables are not available from the vendor. We cannot speak to why the vendor does not provide race variables.***

The matching exercise in Table 2 is useful, but it is not clear how the "bar" of 90% operates for practical decisions. Is 88% that much different to 90% to be notionally pushed aside? How about 80%? It would be useful to have more discussion of the standards you are developing, and the interpretation of the predictivity.

***Author response:***

***We have clarified our interpretation in the text.***

In the concluding paragraph, this sentence stands out: "Future research should continue to monitor and document the availability and accuracy of different types of appended frame data." Availability is the key here. Can you give examples of what other variables should be brought forward? Race, for instance?

***Author response:***

***We are limited by both what the ABS vendors provide on their frames and what we collect in our studies that can be matched to the ABS variables. A way to expand this research would be to assess the availability of variables of interest on the frame, and then design our surveys to collect information in a way that can be matched to the frame variables. This could include race along with other major demographic variables of interest, depending on the nature and subject of the survey and what is available on the ABS frames.***

Reviewer #2: General:
• This study is useful for researchers who deal with quantitative survey data –
particularly on the survey sampling design, data analysis and the interpretation of
the findings.
• It presents the original research. However, there is a level of plagiarism (based on
the ‘Turn It In’, please see results in an attached file).
• Results reported have not been published elsewhere. Although, there are number of
similar published publications, especially the reference # 4 that the authors would
like to follow up with an improvement of the appended data over time (line 96).

***Author response:***

***As noted, the intention of this research was to replicate and follow up on the research presented in reference #4. Any similarities are due to the goal of replication of the research.***

• Mathematics formula and data analysis performed well in an appropriate manner,
but it need references for methods and formulas.

***Author response:***

***We have inserted the requested references.***

• Conclusions are clearly drawn from the results.
• The data analysis of this manuscript was based on the secondary survey data sets,
and appended data from a vendor. The research meets all applicable standards for
the ethnics of research integrity.
Specific:
• Introduction:
The rationale of this study should be strengthened, and research gaps should be
clearly identified. These would be aligned with objectives and methodology.

***Author response:***

***We have addressed this comment in the manuscript.***

• Data and methods:
The authors end up with using three data sets (NHTS, HINTS, ACS), but under in Table
1, the authors provide 2 detail data sets, while the third dataset was not directly use
for data analysis was presented. Why did the authors give the NHES detail? Please
clarify.

***Author response:***

***The reference to NHES in Table 1 is included because we show results using NHES data in Figure 1. The manuscript has been updated to reflect this.***

• Availability and quality of appended demographic information:
o Please define ‘demographic information’ used in this study. Normally,
demographers define demographic variables as age, sex, ethnicity/race of the
population.

***Author response:***

***The items we evaluated have been listed in the manuscript at the beginning of the section entitled “Availability and quality of appended demographic information”.***

o Fig.1 and Fig.2, please label x-axis and y-axis.

***Author response:***

***The figure labels have been updated.***

o Lines 144-145: descriptive, please check its consistency with the figures in
Table 2.

***Author response:***

***This inconsistency has been addressed in the manuscript.***

• Efficiency of using appended demographic variables for oversampling
o “Oversampling” was not mentioned in the rationale- please add argument
why researcher should examine efficiency of using appended data for
oversampling.

***Author response:***

***The manuscript has been updated to address this.***

o Table 3: please clarify why ’18-24 present’ was not highlighted in the table,
while its optimum oversampling rate =2.3 (above 2)
2

***Author response:***

***We did not identify ’18-24 present’ as a variable that could be used efficiently for oversampling so it is not highlighted in the table. We computed optimum oversampling rates for all of the items, but only identified Hispanic origin, Hispanic surname, and 65+ present as having increased the effective yield enough to warrant their use in oversampling. This was based on our general rule of an oversampling scenario where the oversampling rate in the high density stratum is ≥ 1.5 and the increase in effective yield for the subgroup is greater than 10 percent while the overall design effect remains below 1.1.***

o Line 214; according to the results, please clarify why the household income is
the less promising variable.

***Author response:***

***We show low income households as an example of a less promising variable in the figure because the oversampling is not as effective. This is due to the difference between the prevalence in the high-density stratum and low-density stratum being smaller than for the items where oversampling increases the effective yield.***

o The American Community Survey (ACS), please clarify why didn’t the authors
mentioned it at the beginning (in the data and methods session).

***Author response:***

***With the other two surveys (HINTS and NHTS), actual survey responses were compared to the appended variables to evaluate the quality of the appended data and determine the utility of the appended variables for oversampling. With the ACS, we examined the use of aggregate (tract-level) data for oversampling, and compared this to the use of appended variables for oversampling. Thus, the HINTS and NHTS were used to directly examine the quality of the vendor-appended variables at the household level, whereas the ACS (tract-level summaries, not microdata) was considered as a potential alternative to vendor-appended variables for oversampling.***

o Suggest: that the authors add references for each formula, and show a
sample calculation e.g for the first figures in Tables 2 & 3.

***Author response:***

***We have inserted references and sample calculations.***

• Discussion:
The authors should add work more on the strengthen and limitation of this study,
and how it support or contrast with the existing knowledge that was reviewed in the
introduction. At this stage, there is almost no discussion in this session.

***Author response:***

***We have inserted text in the Discussion section on how our findings relate to the findings of other studies.***

• References:
Only 15 references were mentioned, while references related to methods and
calculation should be added

***Author response:***

***We have inserted references related to methods and calculations.***
